# Chemical Composition and Potential Practical Application of 15 Red Algal Species from the White Sea Coast (the Arctic Ocean)

**DOI:** 10.3390/molecules26092489

**Published:** 2021-04-24

**Authors:** Nikolay Yanshin, Aleksandra Kushnareva, Valeriia Lemesheva, Claudia Birkemeyer, Elena Tarakhovskaya

**Affiliations:** 1 Department of Plant Physiology and Biochemistry, Faculty of Biology, St. Petersburg State University, 199034 St. Petersburg, Russia; kolya1256@gmail.com (N.Y.); valeriya.lemesheva@gmail.com (V.L.); 2 N. I. Vavilov Research Institute of Plant Industry, 190000 St. Petersburg, Russia; light6elf@gmail.com; 3 Faculty of Chemistry and Mineralogy, University of Leipzig, 04103 Leipzig, Germany; birkemeyer@chemie.uni-leipzig.de; 4 Vavilov Institute of General Genetics RAS, St. Petersburg Branch, 199034 St. Petersburg, Russia

**Keywords:** red algae, protein, free amino acids, phycoerythrin, heavy metals, White Sea

## Abstract

Though numerous valuable compounds from red algae already experience high demand in medicine, nutrition, and different branches of industry, these organisms are still recognized as an underexploited resource. This study provides a comprehensive characterization of the chemical composition of 15 Arctic red algal species from the perspective of their practical relevance in medicine and the food industry. We show that several virtually unstudied species may be regarded as promising sources of different valuable metabolites and minerals. Thus, several filamentous ceramialean algae (*Ceramium virgatum*, *Polysiphonia stricta*, *Savoiea arctica*) had total protein content of 20–32% of dry weight, which is comparable to or higher than that of already commercially exploited species (*Palmaria palmata*, *Porphyra* sp.). Moreover, ceramialean algae contained high amounts of pigments, macronutrients, and ascorbic acid. *Euthora cristata* (Gigartinales) accumulated free essential amino acids, taurine, pantothenic acid, and floridoside. Thalli of *P. palmata* and *C. virgatum* contained the highest amounts of the nonproteinogenic amino acid β-alanine (9.1 and 3.2 μM g^−1^ DW, respectively). Several red algae tend to accumulate heavy metals; although this may limit their application in the food industry, it makes them promising candidates for phytoremediation or the use as bioindicators.

## 1. Introduction

Marine red macroalgae are known as sources of numerous valuable natural products that currently have high demand in nutrition, medicine, and different branches of industry. In Asian countries, these algae have long been used as food components in a wide array of applications. As algal food products have proved to be highly effective in diets and healthy nutrition, they are being increasingly utilized all over the world [1,2,3]. A number of substances derived from red algae (e.g., polysaccharides, pigments, lectins, amino acids, phenolic compounds) have antioxidant, antibiotic, and anticancer properties [4,5,6]. Moreover, they are efficient in the prevention and correction of diet-related metabolic disorders, such as obesity and type 2 diabetes [7,8]. Besides nutraceutical applications, red algae can be used for the production of biofuel [9] and natural colorants [10] and for bioremediation [11].

Such multiple uses have drawn the attention of scientists all over the world to biochemical studies of these organisms. The most extensively studied red algal compounds are specific cell wall polysaccharides, i.e., sulfated galactans, agar, and carrageenan. Main sources of agar are the genera *Ahnfeltia*, *Gelidium*, and *Gracilaria*. Carrageenan-producing seaweeds mostly belong to the order Gigartinales (*Chondrus*, *Eucheuma*, *Furcellaria*, *Gigartina*, *Kappaphycus*). The content of agar and carrageenan varies from 10 to 60% of dry weight (DW) depending on the species, geographical location, harvesting season, and growth conditions [12]. Both types of galactans are not digested by humans and therefore are used in nutrition as water-soluble dietary fibers and nutraceuticals [1,13].

Red algae have relatively high protein content, up to 30–40% DW, similar to some high-protein vegetables and exceeding the corresponding values for brown and green algae [14,15]. The amino acid composition of red algal protein was thoroughly studied for commercially exploited species, such as *Porphyra tenera* and *Palmaria palmata*. It was shown that essential amino acids constitute up to 46% (*w*/*w*) of the total amino acid fraction of *P. palmata* and that at least a few red algae may be valuable sources of lysine, methionine, valine, and leucine [16,17].

Many edible red algae contain relatively high amounts of both macroelements (K, Ca, Mg, P) and trace elements (Mn, Cu, Zn, Mo). Generally, they contain more minerals compared to land plants and animal products and thus may be effective as a low-calorie food supplement for people in regions deficient in essential minerals [18,19].

Currently, the focus of seaweed biochemical studies is shifting more and more to the detailed analysis of low-molecular-weight metabolites, such as free amino acids (both proteinogenic and nonproteinogenic), sugars and polyols, vitamins, and phenolic compounds. Many of these substances feature multiple biological activities and are extensively elaborated as potential components of therapeutic and cosmeceutical products. From this perspective, red algae are sources of a number of specific compounds such as mycosporine-like amino acids, floridoside, and taurine [20,21]. ‘Omic’ techniques have provided powerful new tools for such studies; in particular, GC-MS-based metabolite profiling allows simultaneous analysis of many primary and secondary low-molecular-weight metabolites. Up to now, metabolomics of marine red algae is not among the well-studied fields of plant physiology; likewise, the investigations are typically focused on commercially exploited species (edible seaweeds and agarophytes). Moreover, Asian species have been studied much more intensively than species inhabiting Arctic seas and North Atlantic, and many red algal species growing abundantly on the shores of the White Sea (e.g., *Euthora cristata*, *Cystoclonium purpureum*, *Ptilota gunneri*) are still very poorly investigated. Meanwhile, studies on seaweed chemical composition within different taxa of red algae and different locations show considerable differences in concentrations of compounds of interest [20,22]. Thus, broadening both the list of studied species and the geographical coverage may reveal new candidates for practical use among red seaweeds.

According to literature data, red algae contribute considerably to the White Sea macroalgal community: 41 species of Rhodophyta, 13 species of Chlorophyta, and 33 species of Phaeophyceae have been registered in the phytocoenoses of the red algal belt in the Kandalaksha and Onega bays [23], and the total list of red algae in the White Sea contains 61 species [24]. The dominating representatives of Rhodophyta in the White Sea coastal waters are *Palmaria palmata*, *Odonthalia dentata*, *Phycodrys rubens*, *Coccotylus brodiei*, *C. truncatus*, *Ahnfeltia plicata*, *Vertebrata fucoides*, *Polysiphonia stricta*, *Lithothamnion glaciale*, *Ptilota gunneri*, *P. serrata*, etc. [23,24,25,26]. Several of these or systematically close species were reported to have multiple biological activities. Representatives of the order Ceramiales (including *Rhodomela confervoides* and different species of *Ceramium*, *Polysiphonia*, and *Odonthalia*) synthesize a plethora of halogenated compounds that exhibit antibacterial, antifungal, anti-inflammatory, anti-allergic, and cytotoxic effects [6,27,28,29]. Several *Ptilota* species were reported to contain different specific lectins [30]. *P. palmata* is known as a source of antioxidants [5] and kainoids (kainic and domoic acids), which are currently considered promising tools in the study of neurophysiological disorders such as epilepsy and Alzheimer’s and Parkinson’s diseases [1,31].

The objective of this study was to compare the chemical composition of 15 red algal species growing in coastal waters of the White Sea. We analyzed the elemental composition, total carbohydrates and protein, photosynthetic pigments, and several low-molecular-weight metabolites.

## 2. Results

### 2.1. General Biochemical Characteristics

The thalli of studied red algae had moisture of 73–89%, except for *A. plicata* (64%) and *C. officinalis* (31%) (Table 1). Due to its calcareous thallus, *C. officinalis* has a relatively high percentage of DW, which should be taken into account during data analysis. Content of total protein varied from 3.4 to 32.3% DW and was especially high in *C. virgatum* and *C. purpureum*. Soluble protein fraction contributed to 37.5–74.9% of the total protein. The maximum level of soluble protein (14.3% DW) was found in the thalli of *S. arctica* (Table 1).

Total carbohydrates contributed to 17.5–56.9% of thallus dry weight, with maximum carbohydrate level found in *F. lumbricalis* and minimum carbohydrate level found in *C. officinalis* (Table 1).

### 2.2. Pigment Content

**Figure 1:**
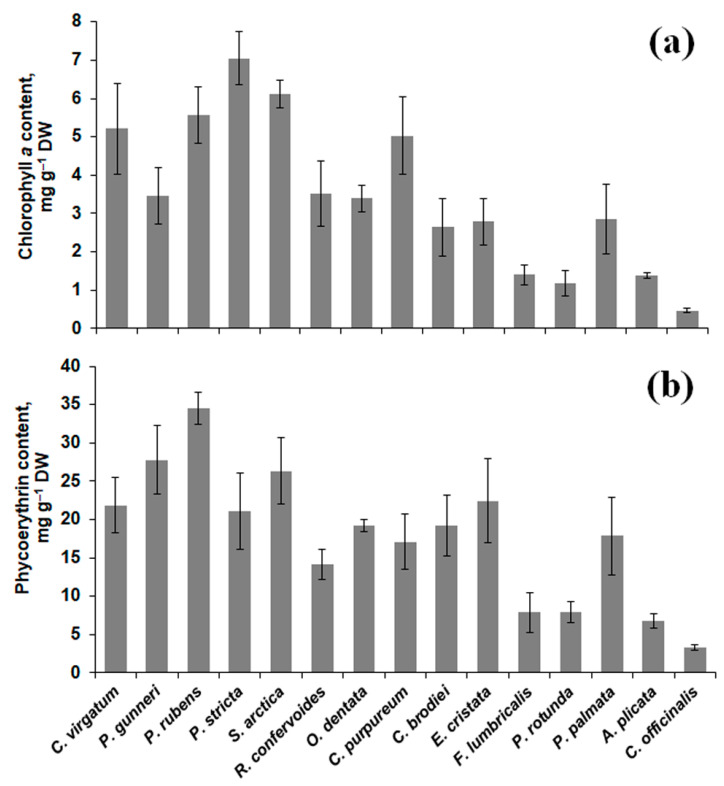
Chlorophyll *a* (**a**) and phycoerythrin (**b**) content in 15 red algal species (means ± SD).

The content of major photosynthetic pigments (chlorophyll *a* and phycoerythrin) in the thalli of different red algal species exhibited considerable variation (Figure 1). Four species (*C. officinalis*, *P. rotunda*, *A. plicata*, and *F. lumbricalis*) contained relatively low amounts of both pigments (~0.5–1.4 mg g^−1^ DW for chlorophyll and ~3–8 mg g^−1^ DW for phycoerythrin). The highest chlorophyll *a* content (7 mg g^−1^ DW) was found in *P. stricta*, and *Ph. rubens* showed the maximum content of phycoerythrin (34.5 mg g^−1^ DW) (Figure 1).

### 2.3. Mineral Composition

Analysis of major element content showed a high correlation (*r* = 0.93–0.99) among different species of the order Ceramiales; on average, these algae contained relatively high levels of K, Ca, Fe, Mg, and P, as well as a low level of S, compared to the other species (Table 2). The only exception was *Ph. rubens*, containing lower amounts of Ca and Fe. On the contrary, representatives of the order Gigartinales contained a relatively high level of S (up to 20.36 mg g^−1^ DW in *E. cristata*) and lower content of other elements. This group of algae did not show a significant correlation, and the most notable outlier was *C. purpureum*, containing higher amounts of K and P (close to the corresponding values for Ceramiales). *P. palmata* contained the maximum level of potassium (~20 mg g^−1^ DW) but was generally poor in macroelements. *Ahnfeltia* showed relatively low content of all analyzed elements. *Corallina* was especially rich in calcium and magnesium deposited in its calcified thalli but contained low amounts of K, P, S, and Fe (Table 2).

Trace metal analysis showed a high correlation among Gigartinales species (*r* = 0.90–0.99) and relatively high variation in the representatives of Ceramiales (Table 3). Several species accumulated particularly high amounts of specific metals: Mn and Co for *O. dentata*, Zn and Mo for *R. confervoides*, Cu for *S. arctica*, and Ni for *C. brodiei. Ahnfeltia* and *Corallina* exhibited the lowest levels of analyzed trace elements. Manganese and cobalt were strongly correlated (*r* = 0.99) in their distribution in thalli of different red algal species (Table 3).

### 2.4. Low-Molecular-Weight Metabolites

**Figure 2:**
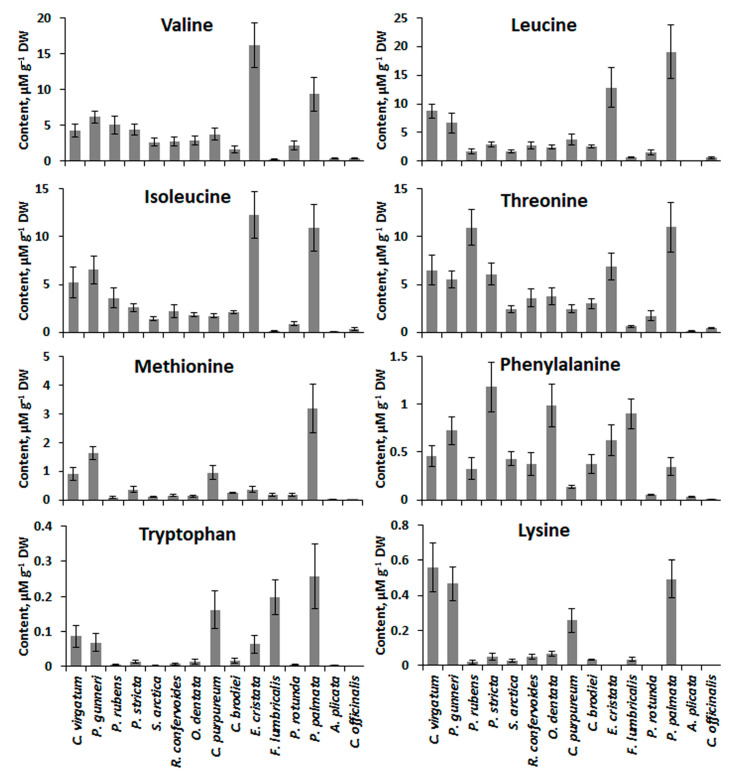
Essential amino acid contents in 15 red algal species (means ± SD).

GC-MS analysis allowed detecting eight free essential amino acids in the thalli of the studied red algae, namely valine, leucine, isoleucine, threonine, methionine, phenylalanine, tryptophan, and lysine. The highest amounts of almost all amino acids were found in two species, *E. cristata* and *P. palmata* (Figure 2). Moreover, several species showed relatively high content of one particular amino acid: lysine for *C. virgatum* and *P. gunneri*, threonine for *P. rubens*, phenylalanine for *P. stricta*, and tryptophan for *F. lumbricalis* and *C. purpureum*.

**Figure 3:**
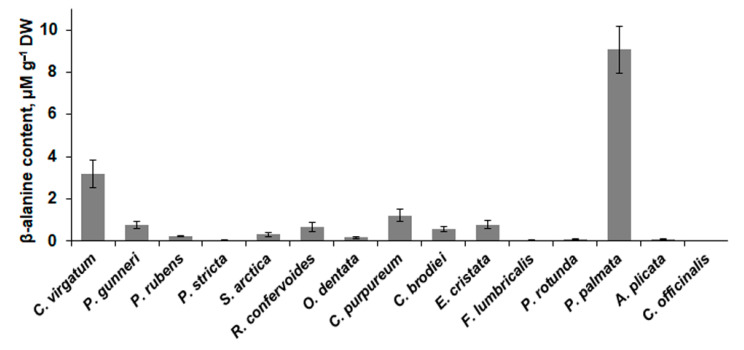
Content of β-alanine in 15 red algal species (means ± SD).

**Figure 4:**
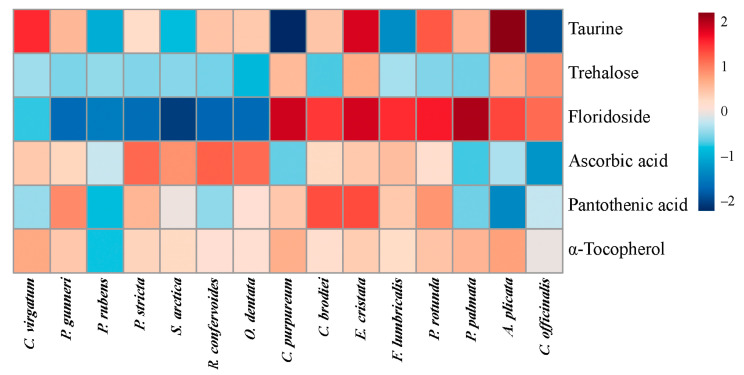
Relative content of several biologically active metabolites in 15 red algal species. Mean values of 5–6 samples are presented on a log_10_ scale.

Several other valuable metabolites specifically accumulated in thalli of several red algae (Figure 3 and Figure 4). *P. palmata* and *C. virgatum* contained the highest amounts of nonproteinogenic amino acid β-alanine (9.1 and 3.2 μM g^−1^ DW, respectively, Figure 3), and *A. plicata*, *E. cristata*, and *C. virgatum* accumulated taurine (Figure 4). All algae except for representatives of the order Ceramiales contained considerable amounts of specific red algal polyol floridoside. The maximum content of this metabolite was found in *P. palmata*, *C. purpureum*, and *E. cristata*. All studied Ceramiales were also relatively poor in trehalose, the highest level of which was found in *C. officinalis*.

Three vitamins (ascorbic acid, pantothenic acid, and α-tocopherol) were detected by GC-MS analysis (Figure 4). Ascorbate mostly accumulated in Ceramiales (maximum in *R. confervoides*), whereas the species containing the highest level of pantothenic acid (*C. brodiei* and *E. cristata*) belonged to Gigartinales. Compared to the other vitamins, tocopherol was generally more evenly distributed among the studied species; its maximum content was found in *A. plicata*, *C. virgatum*, and *C. purpureum* (Figure 4).

## 3. Discussion

Our study provides a comprehensive characterization of the chemical composition of 15 Arctic red algal species from the perspective of their potential practical relevance. We showed that several of the studied species contain different valuable minerals and metabolites.

Currently, seaweeds are regarded as being a cheap alternative source of proteins for human nutrition and animal feeding [22]. Among marine macrophytes, red algae have the highest protein content. According to literature data, different red algae contain from 2.3 (*C. officinalis*) to 47% DW (*Pyropia tenera*) of total protein (reviewed in [15,32]). These values are generally in good accordance with our results (Table 1), and for several well-studied algae, our results confirm data previously obtained on the same species (e.g., 18.3–26.5% DW for *P. palmata* and 2.3–6.9% DW for *C. officinalis*) [33,34,35,36,37]. Most of the protein-rich species in our study belong to the order Ceramiales (*C. virgatum*, *R. confervoides*, *S. arctica*) (Table 1 and Table 4). As none of the species in this order are extensively exploited, information about the biochemical composition of these algae is limited. However, several analyzed ceramialean algae (*Polysiphonia* sp., *Amansia multifida*, *Enantiocladia duperreyi*) were found to have a total protein content close to our values (19.5–31.3% DW) [35,38].

A general problem in utilizing seaweed protein is its limited bioavailability, especially when algae are consumed as sea vegetables, without previous hydrolysis or preparing protein concentrates. Even cooked, algal thalli are relatively tough and difficult to chew. The digestibility of red algal protein is generally estimated as 50–70% (compared to casein, taken to be 100%). It was suggested that seaweeds contain some compounds interfering with protein digestion and further amino acid absorption. Polysaccharides, phenolic compounds, and membrane-associated glycoproteins (including lectins) were regarded as candidates for this role [39,40]. Polysaccharides are usually present in large quantities in algal cells and are supposed to be the main cause of the limited digestibility of the algal proteins [17,34]. However, our data (Table 1) showed that most of the protein-rich red algae have a relatively low content of total carbohydrates (the only meaningful exception was *R. confervoides*). A high amount of phenolics is mostly a problem of brown algae utilization, as they may contain up to 25% DW of specific polyphenols (phlorotannins) [41]. Red seaweeds typically contain lesser amounts of phenolic compounds than brown and green algae [39]. At the same time, it was shown that red algae contain more lectins of different types than the other macrophytes [42]. From this perspective, the water-soluble fraction of protein can be of particular interest for the assessment of the potential nutritional value of red algae, as this fraction is more easily available and hardly contains membrane proteins. It was shown that extraction of lectins from algal material is twice less effective without detergent addition [42]. According to our data, the content of soluble protein fraction in red algae varies considerably and does not always show a correlation with the total protein content. For example, *P. stricta* and *P. palmata* contained similar total amounts of protein, but content of soluble protein in thalli of *P. stricta* was twice higher than in the other species (Table 1). A high total protein level in combination with a high proportion of soluble fraction (more than 50% of total protein) was detected in several representatives of the order Ceramiales (*P. stricta*, *S. arctica*, *R. confervoides*). Thus, we may conclude that some ceramialean algae (especially those having filamentous thalli) can be sources of seaweed protein comparable to, or even more efficient than, broadly used *P. palmata* and different *Porphyra* species.

Besides bioavailability, protein quality is based also on its amino acid composition. Multiple sources of literature data imply that the protein amino acid score and the essential amino acid index in red algae are higher than those in brown and green macrophytes. In particular, the amino acid profile of *P. palmata* protein is very close to that of the standard protein ovalbumin [14,15,34]. Much less information can be found about the profiles of free amino acids in the red algal cells. It was shown that different red algae contain detectable amounts of almost all free essential amino acids, except for tryptophan and sometimes methionine [43,44,45]. However, even for extensively studied species such as *P. palmata*, most papers do not provide quantitative data, reporting just the percentage contribution of different amino acids to the total content of these compounds (e.g., [45,46]). Meanwhile, given the limited digestibility of algal proteins, free amino acids may be regarded as a more easily available source of essential amino acids for human nutrition. In general, the content of essential amino acids in the algae used in our study (Figure 2) is close to the values obtained for the other species, including representatives of Ceramiales, Gigartinales, and Corallinales (e.g., 0.4–7.7 μM g^−1^ DW for valine and 0.1–3.7 μM g^−1^ DW for phenylalanine) [43]. Several species contained considerable amounts of free tryptophan, methionine, isoleucine, and lysine (Figure 2), the amino acids that are often limiting in red algal proteins [32]. Analysis of our data shows that the accumulation of amino acids is strongly species-specific. Two species belonging to different orders, *P. palmata* and *E. cristata*, showed the highest content of most of the essential amino acids (Figure 2). For *P. palmata*, we may suppose that its profile of free amino acids partially reflects the specificity of its protein composition, as leucine, valine, and methionine are known to be well represented in the essential amino acid fraction of *Palmaria* protein [14]. As for *E. cristata*, to our knowledge, this species was analyzed in detail for the first time.

In the cells of the red algae, a considerable proportion of protein is associated with phycobilins, specific photosynthetic pigments, of which phycoerythrin is the most dominant. Currently, phycoerythrin is widely used in biomedicine, due to its antioxidant, anticarcinogenic, neuroprotective, and anti-inflammatory activities; in biochemical techniques (for producing highly sensitive fluorescence probes); and as a natural protein dye for food, cosmetic, and textile industries [10,47]. In this study, several species belonging to Ceramiales (*P. rubens*, *P. gunneri*) showed a phycoerythrin content of about 30 mg g^−1^ DW (up to 48% of soluble protein or 26% of total protein), which is higher than the values measured in crude or purified extracts of commercially exploited *Palmaria* species (9–12% of total protein) (Figure 1b; [48,49]). As the main source of phycobilins is still extraction from the natural producers (red algae and cyanobacteria), broadening the list of used organisms is a highly relevant task.

There was no significant correlation between phycoerythrin and total or soluble protein content (*r* = 0.35–0.4) (Table 1, Figure 1b). Being major light-harvesting pigments in red algal cells, phycobilins are expected to accumulate in the thalli of deep-water species. However, our data imply that a relatively high level of both phycoerythrin and chlorophyll *a* is rather a species-specific characteristic. For example, *P. rubens* and *O. dentata*, two ceramialean species having similar ecology (subtidal seaweeds, frequently growing under the coverage of *Saccharina* fronds), showed significantly different (*p* < 0.05) pigment content. At the same time, intertidal *P. rotunda* and subtidal *F. lumbricalis* contained practically the same amounts of phycoerythrin and chlorophyll *a* (Figure 1). Chlorophylls and their derivatives are currently regarded as valuable constituents of healthy nutrition due to their multiple biological activities including cancer prevention and activation of xenobiotic detoxification processes [50]. An additional benefit of chlorophyll consumption is magnesium uptake. Comparison of different plant sources of Mg showed that this element had higher bioavailability as a constituent of chlorophyll (in green vegetables) than in the form of phytin (in cereals) [51]. According to our data, chlorophyll *a* content in thalli of *P. stricta*, *S. arctica*, and *P. rubens* is comparable to that in green leafy vegetables such as lettuce, spinach, and kale [52,53]. Thus, several ceramialean species can be used as efficient sources of both phycoerythrin and chlorophyll.

Traditionally, seaweeds are valued for their high mineral content, as they contain more essential elements compared to the terrestrial plants [18,19]. The mineral composition of red algae is well documented, and our results are generally in good accordance with literature data [16,33,54,55]. Among the mass of red algal species inhabiting the White Sea coast, the most studied and exploited species is *P. palmata*. Our study shows that this species was relatively poor in major elements, except for potassium. From the perspective of using algal biomass as a source of diverse macroelements, ceramialean algae might be the most promising, as they were demonstrated to have a well-balanced mineral composition (Table 2).

Assessment of potential practical relevance of any seaweeds should necessarily include a survey of trace metal contents in their thalli. Metals such as copper, manganese, zinc, and cobalt are involved in crucial biochemical processes in the human organism, being components of electron-transport chains, vitamins, and enzymes, and algae may be good sources of these elements. On the other hand, heavy metals are toxic in high concentrations, and seaweeds are known as organisms accumulating them, sometimes up to levels a thousand times higher than those of seawater [56,57]. It was reported that there is significant variation in bioaccumulation capacity of different red algal species, and red algae can accumulate several heavy metals more efficiently than other macrophytes [57,58]. In our study, we revealed four species (*O. dentata*, *R. confervoides*, *S. arctica*, and *C. brodiei*) containing especially high amounts of several metals (Table 3), with maximum values close to those observed in experiments with artificially contaminated seawater [56]. Though some of these species (*R. confervoides* and *S. arctica*) exhibited high protein content, their nutritional value may be limited due to their tendency to accumulate zinc and copper. The high capacity for heavy metal accumulation makes these algae candidates for use as bioindicator species. Currently, red algae are not as extensively exploited for biomonitoring in marine ecosystems as brown seaweeds, though several species have already been reported as reliable bioindicators [59,60].

To provide the most comprehensive characteristic of the studied algae, besides quantitative evaluation of protein, carbohydrate, pigment, and mineral content, we also compared the concentrations of several low-molecular-weight metabolites in the algal cells (Figure 3 and Figure 4). Here we focused on the vitamins and other compounds with confirmed biological activity. According to the literature data, red algae contain relatively high amounts of vitamins: 1–11 mg g^−1^ DW of ascorbic acid [61,62], 20–80 μg g^−1^ DW of α-tocopherol [63,64], and 5–150 μg g^−1^ DW of pantothenic acid [64,65], as well as considerable amounts of thiamine, riboflavin, nicotinic acid, and cobalamin [22,65]. Such levels are comparable with the content of these vitamins in traditionally consumed terrestrial vegetables [22,66]. Our results confirmed a considerable variation of ascorbic and pantothenic acid content between different red algal species. Generally, higher amounts of ascorbate were found in the species of the order Ceramiales, whereas pantothenic acid mostly accumulated in the representatives of Gigartinales (Figure 4). Tocopherol was more evenly distributed among the different algal species. Only one type of tocopherol, namely α-tocopherol, featuring the highest antioxidative activity, was detected in all studied algae, which is in accordance with literature data [63].

Recent studies showed that several specific low-molecular-weight carbohydrates, including floridoside and trehalose, have multiple therapeutic effects. Floridoside (α-D-galactopyranosyl-(1–2)-glycerol) is a unique photosynthetic product of red algae, functioning as a storage compound, osmolyte, and antioxidant [67,68]. In clinical investigations, it was reported that floridoside demonstrated strong immunomodulatory effects, activating a complement cascade [69]. Moreover, this substance possesses anti-inflammatory and antioxidative activities [70,71]. Floridoside content in the red algal cells is taxon-specific, with ceramialean algae containing zero or trace amounts of this metabolite [72]. The highest concentrations of floridoside (up to 1 mM g^−1^ DW) were reported from *P. palmata* and several species belonging to the Gigartinales [72,73,74], which corresponds well to our data (Figure 4). Apparently, *C. purpureum* and *E. cristata* may be added to the list of floridoside-rich gigartinalean species along with *Catenella nipae* and *Schottera nicaeensis* [72,73]. Unlike the situation with floridoside, literature data about the occurrence of another low-molecular-weight carbohydrate, trehalose, in red algae are still fragmentary and sometimes ambiguous. This metabolite was detected in several species of Corallinales (including *C. officinalis*), Gigartinales (including *C. purpureum*), Ceramiales, and Batrachospermales [67,72,75]. Recently, it was also found in *Porphyra umbilicalis* [76]. Our results confirm the presence of trehalose in the cells of *C. officinalis* and *C. purpureum*, and we detected similar amounts of this metabolite in *A. plicata* and *E. cristata* (Figure 4). To our knowledge, in the latter two species, trehalose was found for the first time. Currently, trehalose is widely used as a stabilizer of foodstuff, vaccines, and cosmetics, and it is becoming more and more popular in medicine, particularly in ophthalmology [77]. Though red algae apparently contain a relatively low concentration of this metabolite [76], its presence in several species should still be considered regarding the development of technologies of multipurpose use of algal biomass.

Red algae contain a variety of nonproteinogenic amino acids and sulfonic acids, and the synthesis and accumulation of these compounds are strictly species-specific [18]. Several algae analyzed in this study exhibited relatively high content of β-alanine and taurine (Figure 3 and Figure 4). Being a common metabolite of many vascular plants, where it contributes to different stress responses [78], β-alanine has been reported from very few of the red algae [43]. Our data show that most of the studied algal species contained 0.05–1.3 μM g^−1^ DW of this amino acid (Figure 3), which corresponds to the values known from different organs of vascular plants [79]. However, *P. palmata* contained approximately ten times more β-alanine and thus may be regarded as a valuable natural source of this metabolite. Over the past few decades, β-alanine has become one of the most popular sports nutrition ingredients. Being a metabolic precursor of muscle carnosine, it improves athlete performance during high-intensity exercises in strength and power sports [80]. Moreover, β-alanine is used as an efficient and safe alternative to hormone replacement therapy for menopausal women [81].

Taurine is another valuable metabolite accumulating in thalli of several studied algae (*A. plicata*, *E. cristata*, *C. virgatum*) (Figure 4). According to literature data, red algae contain relatively high concentrations of taurine compared to vascular plants and green and brown algae [82,83]. Regarding the species used in our study, it was shown that *P. rubens* and *S. arctica* contained relatively low concentrations of taurine (0.1–0.15 mg g^−1^ DW), while different *Palmaria* species contained approximately 10 mg g^−1^ DW [84,85]. This corresponds to our data, showing the 100-fold difference between taurine contents in *P. rubens* and *P. palmata* (Figure 4). Currently, this amino acid draws the attention of pharmacologists due to its multiple beneficial health effects. It was reported that dietary taurine supplementation may prevent cardiovascular diseases, hypertension, and metabolic syndrome [86]. Taurine-rich red algae may be a valuable addition to the list of sea products (shellfish, prawns, etc.) used to obtain functional foods containing naturally occurring taurine.

## 4. Materials and Methods

### 4.1. Plant Material Collection

Fifteen species of intertidal and subtidal red algae, representing five orders of the class Florideophyceae (Table 4), were collected in the Keret Archipelago (Kandalaksha Bay, White Sea) in June–August 2017–2020. The list of objects includes a majority of red algae inhabiting the region and having considerable biomass [23,24,25,26]. Thalli were collected from the typical habitats of each species (Table 4). To minimize the possible changes in metabolite content induced by the tidal cycle, all intertidal plants were collected at high tide and directly transported to the laboratory in seawater.

### 4.2. Fresh Weight and Dry Weight Determination

For the determination of fresh weight (FW), 8–10 individual thalli (or bundles, for filamentous algae) were blotted with filter paper and weighed. For determination of DW, they were weighed again after drying at 60 °C to the constant weight.

### 4.3. Elemental Analysis

Algal thalli were gently washed with tap water, rinsed with deionized water, and dried for 24 h at 70 °C. One hundred milligrams of dried plant material was ground into fine powder and subjected to a wet-ashing procedure with a mixture of concentrated HNO_3_ and HClO_4_ in the ratio 4:1 (*v*/*v*). After complete digestion, samples were made up to 25 mL with deionized water. The quantitative analysis of the elemental composition was carried out with inductively coupled plasma atomic emission spectroscopy (ICPE-9000, Shimadzu).

### 4.4. Carbohydrate Analysis

Samples of dried plant material (5 mg DW) were ground to fine powder and hydrolyzed in sulfuric acid according to [87]. The content of total carbohydrates in the hydrolysates was determined spectrophotometrically using anthrone reagent [88]. Briefly, 3 mL of anthrone reagent was added to 0.3 mL of the sample (diluted as necessary). The mixture was boiled for 7 min and then immediately cooled in the ice bath. The extinction was measured at 620 nm using SPEKOL 1300 spectrophotometer (Analytik Jena AG, Jena, Germany), and the amount of total carbohydrate was calculated based on a calibration curve with glucose as standard.

### 4.5. Protein Analysis

For extraction of water-soluble proteins, samples of 20 mg FW were homogenized in Tris-HCl buffer (0.02 M, pH 8.3) using a TissueRuptor II homogenizer (QIAGEN, Hilden, Germany). For further extraction of alkali- and detergent-soluble proteins (including membrane proteins), the 3-fold volume of a mixture of 0.5% sodium dodecyl sulfate and 0.5 M NaOH was added to the homogenates, and the samples were boiled for 20 min. Both types of extracts were centrifuged (5000× *g*, 10 min), and the protein content was measured by Lowry assay with bovine serum albumin as standard [89,90]. Herein, the two protein fractions are referred to as ‘soluble’ and ‘total’ protein.

### 4.6. Pigment Analysis

For chlorophyll analysis, fragments of algal thalli (10 mg FW) were ground using a mortar and pestle in 100% acetone with small quantities of Na_2_SO_4_ and NaHCO_3_. Several rounds of extraction were completed with additional acetone until the extract was colorless. The concentration of acetone was adjusted to 90% (in distilled water), and the content of chlorophyll *a* was calculated [91] from data obtained with a SPEKOL 1300 spectrophotometer (Analytik Jena, Jena, Germany).

For determination of phycoerythrin content, fragments of algal thalli (50 mg FW) were homogenized in potassium phosphate buffer (0.1 M, pH 6.8) using a TissueRuptor II homogenizer (QIAGEN, Germany) and left soaking at 4 °C overnight. Then the extracts were centrifuged (5000× *g*, 10 min), and the content of phycoerythrin was determined spectrophotometrically (SPEKOL 1300, Analytik Jena) [92].

### 4.7. Metabolite Analysis

Samples (50 mg FW) of plant material were poured with cold methanol (−25 °C), quickly ground in a precooled mortar and left soaking in 1 mL of cold methanol for extraction. Four hundred microliters of methanol extract was transferred to clean 1.5 mL polypropylene Eppendorf tubes (VWR, Dresden, Germany) and vacuum-dried for subsequent analysis.

Metabolite profiling analyses were carried out according to [93]. Briefly, vacuum-dried extracts were incubated by shaking in methoxyamine hydrochloride (Sigma-Aldrich, Taufkirchen, Germany) solution in pyridine and *N,O*-bis(trimethylsilyl)-trifluoroacetamide (Macherey–Nagel, Düren, Germany). After derivatization, samples were transferred to micro-inserts of glass vials and subjected to GC-MS analysis on an Agilent 6890 gas chromatograph (1 mL/min He flow, splitless mode, injector temperature 250 °C) coupled to an Agilent 5973N quadrupole mass selective detector (Agilent Technologies, Böblingen, Germany) with the standard electron impact ionization. Within each sequence, a mixture of alkanes (C_10_–C_32_) in hexane was measured for the calculation of Kovats retention indices (RIs) [94]. A mixture of authentic standards containing 21 amino acids, 20 sugars and polyols, and 19 organic acids was co-spiked to confirm the identity of expected compounds.

Peak deconvolution was accomplished using AMDIS 2.65. The retention indices were automatically calculated using an AMDIS calibration file containing the batch retention times of each alkane. Golm metabolome database (GMD_20100614_VAR5_ALK, 24.09.2010, [95]) and NIST14 (National Institute of Standards and Technology, Gaithersburg, MD, USA) were used for identification of the peaks based on spectra comparison. Quantitation of metabolites was performed by peak integration of the corresponding extracted ion chromatograms (*m/z* ± 0.5) for representative intense signals at specific retention times using Xcalibur 3.0 software. Where applicable, calibration was performed by the standard addition method using five calibration levels.

### 4.8. Data Analysis

The measurements were performed with 6 to 12 replicates (from different individuals). Excel 2013 (Microsoft, Redmond, WA, USA) and MetaboAnalyst 4.0 Web application (http://www.metaboanalyst.ca, accessed on 24 November 2020) were used for data processing and normalization procedures, creation of figures, and heatmap construction [96]. The metabolomic data processing included peak area normalization to the median of all areas within a chromatogram, generalized logarithm transformation, and range data scaling (mean-centered and divided by the range of each variable). All values are expressed as means and standard deviations.

## 5. Conclusions

In this study, we compared the chemical composition of 15 red algal species inhabiting the Russian Arctic. The studied algae showed dramatic differences in concentration of proteins, carbohydrates, minerals, pigments, and several low-molecular-weight metabolites. For several parameters (protein, major elements, floridoside) the values were consistent within the orders of Rhodophyta, whereas the accumulation of free amino acids and vitamins was strongly species-specific. The results allow distinguishing several red algal species not studied in detail earlier. These algae showed similar or higher values of all estimated parameters compared to widely commercially exploited species *P. palmata*. Among the most promising species are representatives of the order Ceramiales, demonstrating a high content of protein (*C. virgatum*, *P. stricta*, *R. confervoides*, *S. arctica*), minerals, pigments (*P. rubens*, *P. gunneri*), and ascorbic acid (*P. stricta*, *R. confervoides*). Gigartinalean alga *E. cristata* featured a relatively high content of free essential amino acids, taurine, pantothenic acid, and floridoside. Several species showed a tendency to accumulate heavy metals, which makes them candidates for use as bioindicator species.

## Figures and Tables

**Table 1:** Moisture, protein, and carbohydrate content in 15 red algal species (means ± SD).

Species	Moisture, %	Protein, % DW		Total Carbohydrates, % DW
		Soluble Protein	Total Protein	
*Ceramium virgatum*	86.77 ± 3.13	7.79 ± 1.90	32.25 ± 2.18	32.33 ± 4.31
*Ptilota gunneri*	82.90 ± 3.05	6.69 ± 0.36	8.92 ± 0.55	40.26 ± 4.06
*Phycodrys rubens*	76.71 ± 2.66	7.16 ± 0.72	13.21 ± 1.32	40.83 ± 3.00
*Polysiphonia stricta*	86.38 ± 4.32	11.63 ± 1.92	18.58 ± 4.18	29.38 ± 2.25
*Savoiea arctica*	81.93 ± 2.16	14.28 ± 2.45	24.95 ± 4.71	32.50 ± 8.19
*Rhodomela confervoides*	80.87 ± 2.98	12.15 ± 0.78	24.26 ± 1.26	44.38 ± 3.18
*Odonthalia dentata*	79.64 ± 2.40	6.43 ± 0.44	14.78 ± 2.64	38.09 ± 4.78
*Cystoclonium purpureum*	89.42 ± 3.71	11.88 ± 1.24	27.10 ± 2.36	27.24 ± 7.36
*Coccotylus brodiei*	72.53 ± 3.18	6.96 ± 0.74	15.87 ± 1.38	33.27 ± 3.59
*Euthora cristata*	84.24 ± 2.56	6.52 ± 0.62	13.06 ± 2.47	41.59 ± 4.50
*Furcellaria lumbricalis*	77.20 ± 2.02	10.45 ± 1.66	15.98 ± 1.44	56.92 ± 4.49
*Polyides rotunda*	73.26 ± 4.01	3.46 ± 0.54	9.23 ± 1.53	33.02 ± 0.95
*Palmaria palmata*	88.21 ± 2.65	5.60 ± 0.57	19.94 ± 3.58	24.06 ± 4.17
*Ahnfeltia plicata*	64.03 ± 2.01	3.02 ± 0.39	5.57 ± 1.14	41.27 ± 3.63
*Corallina officinalis*	31.28 ± 1.90	1.39 ± 0.23	3.37 ± 0.46	17.46 ± 3.76

**Table 2:** Major elements in 15 red algal species (means ± SD).

Species	Mineral Content, mg g^−1^ DW					
	K	Ca	Mg	P	S	Fe
*Ceramium virgatum*	10.18 ± 0.15	5.78 ± 0.83	6.47 ± 0.44	2.52 ± 0.01	6.99 ± 0.09	0.32 ± 0.02
*Ptilota gunneri*	8.72 ± 0.21	6.63 ± 0.63	3.34 ± 0.28	2.42 ± 0.03	5.08 ± 0.25	0.43 ± 0.03
*Phycodrys rubens*	1.42 ± 0.26	6.66 ± 0.92	8.55 ± 0.63	2.68 ± 0.08	11.92 ± 0.75	0.25 ± 0.03
*Polysiphonia stricta*	10.33 ± 0.35	4.49 ± 0.62	3.50 ± 0.08	1.22 ± 0.03	10.00 ± 0.32	0.72 ± 0.16
*Savoiea arctica*	8.56 ± 0.24	4.19 ± 0.60	2.57 ± 0.10	1.12 ± 0.06	8.93 ± 0.67	1.84 ± 0.59
*Rhodomela confervoides*	14.32 ± 0.04	5.44 ± 0.05	5.03 ± 0.02	2.45 ± 0.01	12.81 ± 0.03	0.53 ± 0.11
*Odonthalia dentata*	10.70 ± 0.15	4.07 ± 0.33	2.86 ± 0.13	1.25 ± 0.03	9.25 ± 0.39	1.34 ± 0.07
*Cystoclonium purpureum*	14.99 ± 0.08	3.95 ± 0.20	4.61 ± 0.05	2.28 ± 0.08	6.96 ± 0.05	0.43 ± 0.06
*Coccotylus brodiei*	7.05 ± 0.35	4.50 ± 0.16	3.13 ± 0.12	1.38 ± 0.04	15.36 ± 0.89	0.39 ± 0.06
*Euthora cristata*	1.85 ± 0.22	6.73 ± 0.55	4.83 ± 0.30	2.37 ± 0.01	20.36 ± 0.26	0.39 ± 0.09
*Furcellaria lumbricalis*	6.09 ± 0.12	2.18 ± 0.10	5.55 ± 0.12	1.29 ± 0.02	13.44 ± 0.28	0.43 ± 0.02
*Polyides rotunda*	4.86 ± 0.24	3.69 ± 0.16	3.15 ± 0.10	0.99 ± 0.01	8.61 ± 0.19	0.29 ± 0.04
*Palmaria palmata*	19.99 ± 2.00	1.61 ± 0.13	0.92 ± 0.11	2.12 ± 0.05	1.73 ± 0.01	0.72 ± 0.08
*Ahnfeltia plicata*	3.47 ± 0.35	6.54 ± 0.40	2.05 ± 0.09	1.41 ± 0.02	4.37 ± 0.18	0.26 ± 0.03
*Corallina officinalis*	1.57 ± 0.21	309.7 ± 9.7	14.7 ± 0.71	0.56 ± 0.02	4.03 ± 0.21	0.28 ± 0.09

**Table 3:** Trace elements in 15 red algal species (means ± SD).

Species	Mineral Content, μg g^−1^ DW					
	Mn	Cu	Zn	Mo	Co	Ni
*Ceramium virgatum*	83.64 ± 5.16	6.79 ± 1.63	59.15 ± 5.52	1.41 ± 0.08	<0.1	3.71 ± 0.25
*Ptilota gunneri*	210.4 ± 3.66	9.64 ± 0.94	75.66 ± 5.51	0.53 ± 0.07	1.09 ± 0.39	19.99 ± 4.02
*Phycodrys rubens*	43.10 ± 16.63	19.76 ± 2.31	100.3 ± 6.09	1.44 ± 0.23	<0.1	9.36 ± 0.28
*Polysiphonia stricta*	279.1 ± 12.16	26.97 ± 2.64	86.33 ± 1.67	1.84 ± 0.10	1.35 ± 0.18	6.35 ± 0.39
*Savoiea arctica*	63.62 ± 8.81	42.54 ± 3.02	235.8 ± 8.38	1.23 ± 0.26	0.19 ± 0.03	10.57 ± 0.82
*Rhodomela confervoides*	4.72 ± 0.50	15.40 ± 0.73	286.7 ± 0.72	4.33 ± 0.19	0.08 ± 0.01	7.25 ± 0.81
*Odonthalia dentata*	1751.2 ± 56.2	30.30 ± 0.65	64.10 ± 2.96	0.70 ± 0.10	14.05 ± 0.30	14.82 ± 0.17
*Cystoclonium purpureum*	778.1 ± 31.28	7.39 ± 0.39	154.8 ± 8.67	2.00 ± 0.39	6.11 ± 0.32	9.78 ± 0.70
*Coccotylus brodiei*	597.0 ± 53.06	6.70 ± 0.08	67.85 ± 4.97	0.76 ± 0.03	3.69 ± 0.20	45.32 ± 3.62
*Euthora cristata*	132.9 ± 16.18	14.62 ± 0.26	75.70 ± 2.50	2.16 ± 0.55	0.76 ± 0.01	11.59 ± 0.75
*Furcellaria lumbricalis*	321.4 ± 14.35	7.14 ± 0.24	34.93 ± 2.17	0.52 ± 0.02	1.25 ± 0.16	5.14 ± 0.47
*Polyides rotunda*	157.2 ± 12.31	4.40 ± 0.88	34.80 ± 1.64	0.37 ± 0.03	1.02 ± 0.11	11.16 ± 0.45
*Palmaria palmata*	81.64 ± 10.19	11.06 ± 0.46	98.56 ± 9.97	0.52 ± 0.02	0.43 ± 0.02	11.17 ± 0.36
*Ahnfeltia plicata*	56.72 ± 3.27	4.63 ± 0.40	34.78 ± 1.29	0.56 ± 0.07	0.34 ± 0.01	5.79 ± 0.40
*Corallina officinalis*	171.1 ± 5.87	3.60 ± 0.21	31.05 ± 3.71	0.06 ± 0.01	0.30 ± 0.04	3.68 ± 0.18

**Table 4:** Red algal species used as objects in this study and their typical habitat on shores of the Kandalaksha Bay, White Sea.

Order	Species	Typical Habitat
Ceramiales	*Ceramium virgatum*	Low intertidal–subtidal: exposed rocky shores
	*Ptilota gunneri*	Subtidal: rocks, *Saccharina* holdfasts
	*Phycodrys rubens*	Subtidal: rocks, *Saccharina* stipes and holdfasts
	*Polysiphonia stricta*	Subtidal: rocks, *Saccharina* stipes and holdfasts
	*Savoiea arctica*	Subtidal: fucoid beds, *Saccharina* holdfasts
	*Rhodomela confervoides*	Low intertidal–subtidal: rock pools, fucoid beds, *Saccharina* holdfasts
	*Odonthalia dentata*	Subtidal: rocks, *Saccharina* stipes and holdfasts
Gigartinales	*Cystoclonium purpureum*	Subtidal: rocky shores, frequently growing on *Ahnfeltia* thalli
	*Coccotylus brodiei*	Subtidal: rocks, *Saccharina* stipes and holdfasts
	*Euthora cristata*	Subtidal: rocks, *Saccharina* stipes and holdfasts
	*Furcellaria lumbricalis*	Subtidal: rocks, *Saccharina* holdfasts
	*Polyides rotunda*	Low intertidal–subtidal: rock pools, sandy and rocky bottom
Palmariales	*Palmaria palmata*	Mid-intertidal–subtidal: rock pools, *Saccharina* stipes
Ahnfeltiales	*Ahnfeltia plicata*	Subtidal: rocky shores
Corallinales	*Corallina officinalis*	Subtidal: rocks, fucoid beds, *Saccharina* stipes and holdfasts

## Data Availability

Data associated with the paper are available from the authors upon reasonable request.
